# The epidemiology of hospital treated traumatic brain injury in Scotland

**DOI:** 10.1186/1471-2377-14-2

**Published:** 2014-01-06

**Authors:** Tara Shivaji, Andrew Lee, Nadine Dougall, Thomas McMillan, Cameron Stark

**Affiliations:** 1 Department of Public Health NHS Highland, Assynt House, Beechwood Park, Inverness IV2 3BW, Scotland; 2 Information Services Division, NHS National Services Scotland, Gyle Square, 1 South Gyle Crescent, Edinburgh EH12 9 EB, Scotland; 3 NMAHP Research Unit, School of Nursing, Midwifery & Health, Unit 13 Scion House, University of Stirling, Stirling FK9 4NF, Scotland; 4 Institute of Health and Wellbeing, College of Medical, Veterinary and Life Sciences, University of Glasgow, Gartnavel Royal Hospital, 1055 Great Western Road, Glasgow G12 0XH, Scotland; 5 Centre for Rural Health, University of Aberdeen, Centre for Health Sciences, Old Perth Road, Inverness IV2 3JH, Scotland

**Keywords:** Traumatic brain injury, Accidental falls, Patient admissions, Epidemiology, Scotland, Trends

## Abstract

**Background:**

Traumatic Brain Injury (TBI) is an important global public health problem made all the more important by the increased likelihood of disability following a hospital admission for TBI. Understanding those groups most at risk will help inform interventions designed to prevent causes of TBI, such as falls prevention measures. This study identifies the rate of hospitalisation episodes of TBI in Scotland, explores causes of TBI admissions, and trends in hospitalisation episodes by age and gender over a twelve year period using routinely collected hospital data.

**Methods:**

A retrospective analysis of routine hospital episode data identified records relating to TBI for the twelve years between 1998 and 2009. Descriptive and joinpoint regression analysis were used, average annual percentage changes (AAPC) and annual percentage change (APC) in rates were calculated.

**Results:**

Between 1998 and 2009 there were 208,195 recorded episodes of continuous hospital care in Scotland as a result of TBI. Almost half (47%) of all TBIs were the result of falls, with marked peaks observed in the very young and the oldest groups. The AAPC of hospitalization episode rates over the study period for boys and girls aged 0-14 were -4.9% (95% CI -3.5 to-6.3) and -4.7% (95% CI -2.6 to -6.8) respectively. This reduction was not observed in older age groups. In women aged 65 and over there was an APC of 3.9% (95% CI 1.2 to 6.6) between 2004 and 2009.

**Conclusions:**

Hospitalisation for TBI is relatively common in Scotland. The rise in the age-adjusted rate of hospitalisation episodes observed in older people indicates that reduction of TBI should be a public health priority in countries with an ageing population. Public health interventions such as falls prevention measures are well advised and evaluations of such interventions should consider including TBI hospitalisation as an alternative or supplementary outcome measure to fractured neck of femur. Further research is needed to advance understanding of the associations of risk factors with increased incidence of TBI hospital episodes in the elderly population.

## Background

Traumatic brain injuries (TBI) represent a significant public health problem in the UK and across the world. It is estimated that across Europe there is an average incidence of approximately 235 per 100,000, with most countries experiencing an incidence in the range of 150–300 / 100,000 per year [1].

The severity of TBI is often classified using the Glasgow Coma Scale. Patients with a score of 8 or less are classed as severe, 9–12 are moderate and scores of 13–15 are mild [2]; 90% of injuries are classified as mild [3]. Mild injuries can be associated with significant impairment, disability and long term morbidity [4-6]. Hospitalisation due to TBI is associated with an increased risk of epilepsy, depression [7], cognitive impairment [8] and death [6,9].

The prevalence of disability after hospitalisation for TBI has been reported to be particularly high in Scotland [5] and an improved understanding of the epidemiology of TBI in Scotland is required so that measures to prevent and treat the burden of morbidity can be developed and evaluated [10].

The National Health Service (NHS) in Scotland is publically funded, and provides an integrated care system, including primary care, accident and emergency and in-patient treatment. Almost all brain injury admissions will be to NHS hospitals. This study aims to estimate the rate of continuous in-patient hospital episodes as a result of TBI, and explore trends observed of TBI hospitalisations in recent years in order to help support service planning. This will provide hospital epidemiological estimates to assist in identification of particular groups deemed to be at high risk and thereby providing comparison measures against which future population based interventions targeted at these groups can be evaluated.

## Methods

This study capitalises on Scotland’s capability as a laboratory for data linkage research and makes use of the unique community health index (CHI) number assigned to each person which can be used to link hospital episodes forwards and backwards in time. A retrospective analysis was undertaken of existing routine hospital data related to TBI recorded in Scotland from 1998 until 2009, contained in the Scottish Morbidity Record (SMR01) data-set. SMR01 includes all inpatients and day cases discharged from hospitals across Scotland, apart from those discharged from psychiatric or obstetric units. Individual discharge records were combined during analysis to identify periods of continuous hospital care so that the combined records for each person from admission with TBI to discharge home were counted as one continuous episode; for instance a transfer from the care of one Consultant to another, or a discharge from a general hospital setting to rehabilitation facility was counted as one episode of care. It is possible that the same individual could have been admitted more than once, so the study is therefore a description of continuous care episodes rather than the number of affected individuals.

There is no single system of Accident and Emergency attendance recording in Scotland, and so no national Accident and Emergency data are available. This study was, therefore, confined to continuous in-patient episodes and day case admissions recorded in SMR01.

The SMR01 contains demographic, diagnostic and procedural information, and for the study time span was coded according to the International Classification of Diseases 10th Revision (ICD-10). Filters were used to identify the relevant ICD 10 codes in any recorded diagnostic position. Population based surveillance relies on the elaboration of a clear case definition; this has been a limitation of some recent epidemiological descriptions of TBI [1]. In the US, the Centre for Disease Control and Prevention (CDC) developed a multi-state TBI surveillance system to establish the magnitude and impact of TBI on its population. To increase comparability with other work, the codes used in this study were chosen to correspond with those suggested by the CDC for epidemiological surveillance [11], (Table 1).

**Table 1:** ICD 10 codes used to identify cases of traumatic brain injury

**Description of injury**	**Code**
Open wound of the head	S01.0, S01.9
Fracture of skull and facial bones	S02.0, S02.1, S02.3, S02.7, S02.9
Injury to optic nerve and pathways	S04.0
Intracranial injury	S06.0, S06.9
Crushing injury of head	S07.0, S07.1, S07.8. S07.9
Other unspecified injuries of head	S09.7, S09.9
Open wounds involving head with neck	T01.0
Fractures involving head with neck	T02.0
Crushing injuries involving head with neck	T04.0
Injuries of brain and cranial nerve with injuries of nerves and spinal cord at neck level	T06.0
Sequelae of injuries of head	T90.1, T90.2, T90.4, T90.5, T90.8, T90.9

The CDC definition of TBI is as follows:

An occurrence of an injury to the head with one or more of the following attributable to the head injury

Decreased level of consciousness

Amnesia

Skull fracture

A neurological, neurophysiological or intracranial lesion

OR

An occurrence of death from trauma with head injury listed in the sequence of conditions leading to death.

Rates of hospitalisation episodes for traumatic brain injury for individual years were calculated using national population estimates stratified by gender and age group; population data were obtained from the National Records of Scotland for that year or period.

For the data analysis, Joinpoint Regression Analysis (version 3.5) was used to assess changes in trend of the age standardised annual rates of hospitalisation. Joinpoint software has a specific application for trend analysis where data do not appear to be represented by a single regression slope, but instead are represented by a series of linear segments connected at ‘joinpoints’ or ‘elbows’ in the data with joinpoints being typically marked in time by years where the trend appears to have altered. Joinpoint is used extensively in the analysis of cancer trend data and is freely available to download for use from the US National Cancer Institute website (http://surveillance.cancer.gov/joinpoint/). Although Joinpoint has primarily been used in cancer research, it has crossed over into widespread use in trend analysis in other areas, for example stroke [12], heart disease [13], chronic obstructive pulmonary disorder [14] and suicide [15,16]. The software uses trend data in fitting the simplest joinpoint model permitted and tests whether an apparent change in trend is statistically significant. A Monte Carlo permutation method was used to test for significance, a powerful means for testing the trend observed [17]. The loglinear regression model was selected to detect changes in age-standardised hospitalisation incidence and mortality rates within the timespan 1998–2009. Annual percentage changes with 95% confidence intervals (95% CI) were also estimated. Weighted least squares method was selected to handle heteroschedastic (non-constant) variance using the standard error of each age-standardised rate. A grid search was used to identify a maximum of 2 joinpoints against a null hypothesis of no joinpoints. A maximum of 2 joinpoints was selected with the condition of at least four years required between joinpoints and three years from either end of the timespan.

This study was conducted as part of routine service evaluation for the Scottish Acquired Brain Injury Network (SABIN) and did not require ethical review. The study used anonymised, routinely collected data which was held by the Information and Statistics Division (ISD) of NHS Scotland. Approval from the Privacy Advisory Committee at ISD was not required, as no new linkage was required.

## Results

In the twelve year period under study, there were 208,195 continuous stays in Scottish Hospitals where the recorded diagnosis was compatible with having sustained a traumatic brain injury. The age standardised average rate of hospitalisation over this period was highest in people aged less than 35 years with a further peak observed in people aged over 65. Men accounted for 70% of recorded hospital episodes in the study time span.

The trends in annual rates of hospitalisation were further examined using joinpoint analysis (Table 2). For both men and women there were two occasions during the study time span where a change in trend occurred, indicated by the statistically significant finding of two joinpoints for each group. In men, we observed that the two trend changes occurred in 2002 (95% CI 2000 to 2004) and again in 2005 (95% CI 2003 to 2007). In women, the two trend changes occurred in 2001 (95% CI 2000 to 2003) and in 2004 (95% CI 2003 to 2007).

**Table 2:** Trends in age standardised annual rates of hospitalisation episodes, of TBI by gender, Scotland, 1998 - 2009

**Group**	**Number of joinpoints**	**P value **^**1**^	**Time period**	**APC**^**2**^	**95% CI**^**3**^
Men	2 joinpoints	0.0091	1998-2002	0.6	4.2 to 5.7
			2002-2005	−7.9	−21.9 to 8.6
			2005- 2009	1.6	−3.6 to 7.1
Women	2 joinpoints	0.001	1998-2001	4.2	0.9 to 7.5
			2001-2004	−5.9	−11.6 to 0.1
			2004 - 2009	1.0	−0.4 to 2.5

^1^The p value relates to likelihood of that number of joinpoints compared to the null hypothesis of zero expected joinpoints.

^2^Annual Percentage Change.

^3^95% Confidence Interval for the APC estimates.

Annual trends were further analysed by age group, using age-standardised rates. In both males and females aged 0–14 years there was a reduction in continuous hospital episodes over the whole study period. In boys the Average Annual Percentage Change (AAPC) from 2005 onwards was −4.9% (95% CI −6.3 to −3.5%; and see Table 3). This translated to 871 fewer hospital episodes in 2009 compared to 2005. In girls, over the same period, the AAPC was statistically significantly reduced by −4.7% (95% CI −6.8 to −2.6; and see Table 3), representing a reduction of 227 hospital episodes during the same period.

**Table 3:** Age specific trends in annual rates of hospitalisation episodes for TBI, Scotland, 1998 to 2009

**Males**					
**Age group**	**No. of joinpoints**	**P value**^**1**^	**Time period**	**APC**^**2**^	**95% CI**^**3**^
Age 0-14	1 join point	0.02	1998-2002	0.7	−2.9 to 4.5
			2002- 2009	−4.9	−6.6 to −3.2
Age 15-34	0 joinpoints		1998-2009	−3.8	−5.2 to −2.2
Age 35-64	2 joinpoints	0.01	1998-2002	1.0	−4.2 to 6.5
			2002-2005	−7.8	−22.5 to 9.5
			2005-2009	3.1	−2.4 to 9.0
Age 65+	0 Joinpoints		1998-2009	0.9	−0.1 to 1.9
**Females**					
**Age group**	**No. of joinpoints**	**P value**^**1**^	**Time period**	**APC**^**2**^	**95% CI**^**3**^
Age 0-14	1 joinpoint	0.023	1998-2003	−0.2	−3.2 to 3
			2003 -2009	−4.7	−7.2 to −2.1
Age 15-34	2 joinpoints	0.001	1998-2001	4.5	−3.8 to 3.5
			2001-2004	−12.4	−26.4 to 4.4
			2004- 2009	1.7	−2.5 to 6.1
Age 35-64	2 joinpoints	0.004	1998-2001	5.4	−6.2 to 18.4
			2001 -2004	−7.6	−17.5 to 3.6
			2004-2009	4.5	−3.0 to 12.6
Age 65+	2 joinpoints	0.05	1998-2001	7.3	0.7 to 14.4
			2001-2004	−2.8	−13.9 to 9.8
			2004-2009	3.9	1.2 to 6.6

^1^The p value relates to likelihood of that number of joinpoints compared to the null hypothesis of zero expected joinpoints.

^2^Annual Percentage Change.

^3^95% Confidence Interval for the APC estimates.

In contrast to the reductions observed in childhood hospital episodes, for men and women aged over 65 years an overall increase in age standardised rates was observed during the study period. In men, there was a non-significant AAPC increase of 0.9% (95% CI −0.1 to 1.9) for the study period. For women aged 65 years and over there were two statistically significant joinpoints (p = .05) in 2001 and 2004, corresponding to an observed increase in Annual Percentage Change of 7.3% (95% CI 0.7 to 14.4) for 1998–2001, followed by a non-significant reduction in APC of −2.8% (−13.0 to 9.8) during 2001–04 and then thereafter a further significant increase in APC of 3.9% (95% CI 1.2 to 6.6) to 2009 (all Table 3).

**Figure 1:**
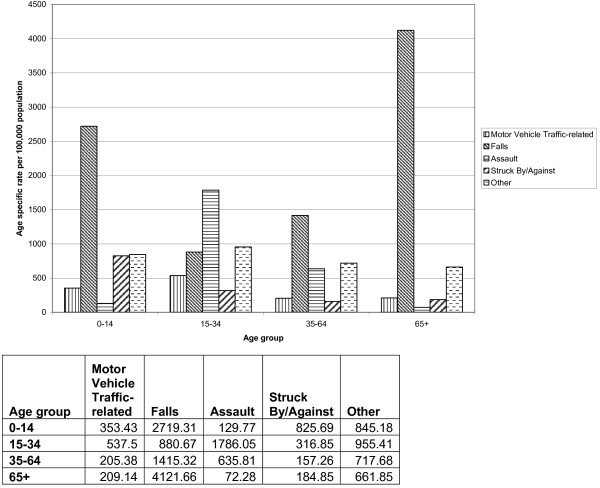
Cause of injury for each continuous episode of care, age specific rates per 100,000 for men and women by age group covering the period from 1998–2009.

With respect to the causes of TBI admission, by far the leading cause was falls with 47% of total continuous care episodes being attributed to this category. Next were assaults accounting for 18% of continuous care episodes overall, however in the age groups 15–34, violent causes are the predominant cause of injury, accounting for 40% of hospitalisations (Figure 1).

## Discussion

In Scotland in 2009, the rate of continuous hospital stays as a result of TBI was 446.4 / 100,000 (95% CI 438.2 – 454.7) in men and 194.8 / 100,000 (95% CI 189.5-200.1) in women. The rate of TBI related hospital admissions for both genders recorded in Scotland appears higher than its European neighbours which have reported rates of 150–300 / 100,000 per year [1], and appears more similar to the overall annual incidence of TBI in the USA of 506.4 / 100,000 population. Direct comparison of the results of this study with the US is limited; in the US surveillance systems, morbidity data is coded by ICD 9 not ICD 10 [18]. In keeping with other studies [1], more men than women were admitted to hospital.

The overall reduction in the rates of TBI-related hospitalisation observed in this Scottish study in the age groups 0–14, 15–34 and 35–64 have also been observed in other countries. A 51% decline in rates of admission for TBI was observed between 1980 and 1995 in the USA, with correspondingly large decreases being noted in rates of hospitalisation in children and young people [19]. A Canadian study also reported a decrease in TBI-related admissions in young people, but as this was accompanied by a decrease in the proportion of mild injuries, changes to treatment patterns may have contributed [20].

The decrease in hospitalisations for TBI in Scotland was not mirrored by a corresponding increase in death rates amongst those who did not reach hospital. Mortality from external causes has remained largely stable over the period under study with an average rate of 65 deaths / 100,000 population per year [21]. This finding is similar to that in other countries where decreases in the mortality rate have also occurred [19,22].

It is unlikely that the observed decrease in mortality reported elsewhere [21] is explained by a downward national trend in the numbers of people admitted to and subsequently discharged from hospital. These trends have remained stable over the past 10 years across the UK [23]. During the study period, children’s services in the UK have seen an increase of 41% in general admissions to short stay services [24], however admissions for TBI in Scotland have decreased. The observed reduction in rates of hospitalisation due to TBI in children has been attributed to effective prevention measures including; legislation for child restraints in cars, improvements in the safety and design of children’s equipment as well as a reduction in the number of child pedestrians and cyclists [25].

Between 1988 and 1998, rates of hospitalisation due to TBI in New Zealand also fell in a pattern which is similar to that observed in Scotland. This reduction was attributed to changes in the diagnosis and management of traumatic brain injury, particularly in mild injuries [22]. During the study period, guidelines for the management of head injury were published in England and in Scotland [26]. Audits of adherence to head injury guidelines are available for England and reveal a low adherence [27] and no corresponding detectable decrease in admissions [28]. It is therefore difficult to estimate the impact of the publication and update of these management guidelines on the incidence of hospitalised TBI.

This study is the first in Scotland to report on the pattern of increase in falls observed in women aged over 65 years and resulting in hospitalisation. This is in agreement with that reported elsewhere, where corresponding rises in rates of hospitalisation in people aged 65 and over has been described in Finland [29] and the United States [30,31] and Australia [32]. Falls accounted for most (79%) admissions in people aged 65 and over in this Scottish study cohort. Older people who sustained a TBI through falling have been reported to be more likely to have co-morbid conditions [30]. Older age is recognised as an independent predictor of poor outcome from hospitalisation [33], and in one study the highest case fatality rates after hospitalisation for TBI were in older people [34].

As the population ages the incidence of TBI sustained from falls is emerging as one of substantial public health importance. Falls prevention interventions could be of value in limiting this morbidity and mortality. Although high quality evidence is limited, a Cochrane review concluded that population-based interventions may be of value in preventing falls in people over 65 [35]. At present there are a number of initiatives across Scotland directed at preventing falls in identified high-risk groups. It would be of value for these initiatives, when assessing their effectiveness, to consider all fall-related hospitalisations [36] instead of limiting the outcome variable to fractured neck of femur. In contrast to falls, TBI-related injuries sustained as a result of violence were a common cause of traumatic brain injury in young men in Scotland, and have a particular association with socioeconomic deprivation [37].

Limitations of the study methodology require consideration in the interpretation of these findings. One limitation is concerned with the underreporting of less severe categories of TBI. As is common with all routine hospital datasets, the SMR01 hospital dataset only identifies the individuals who seek care. People who sustain injuries during sporting activities, as a result of domestic violence, whilst under the influence of alcohol or drugs are less likely to seek care of any form [18]. Not all people who present to accident and emergency departments are onwardly admitted to hospital. In the USA, only 20% of people who presented to hospital were subsequently formally admitted [18]. The remainder attended emergency departments [38], primary care or private facilities. An estimated 6.6% of all emergency department attendances at NHS hospitals in the UK are because of traumatic brain injury related complaints [39]. The majority of those not admitted have sustained a mild injury [40], suggesting that the effects of underestimation are likely to be more significant for less severe injuries [22].

The interpretation of results must also consider the data quality as a limitation, and in particular the accuracy of the coding process for injuries where inconsistencies have been demonstrated before in the use of codes, particularly for minor injuries [41]. It is also important to note that the figures are for continuous hospitalisation episodes after TBI, and it is possible for one individual to have had more than one admission.

## Conclusions

This study is the first to report on the pattern of increase in falls observed in women aged over 65 years in Scotland, and identifies several avenues for further research. Further examination into the rise in annual rates seen in the over 65’s would be of value to identify associated reasons and risk factors for this rise. Of particular interest is the identification of any falls category which is amenable to preventative measures and whether the target group is included in any current or future interventions directed towards falls prevention.

Violence continues to be an important cause of brain injury in younger men, and describing geographical patterning would be valuable, as this would help to target prevention activities. Understanding the clinical outcome of hospitalised cases of TBI is an important next step in understanding the disease burden and the availability in Scotland of linked data sets is one potential tool in progressing with this endeavour.

## Abbreviations

TBI: Traumatic brain injury; CHI: Community health index; SMR: Scottish morbidity record; ICD: International Classification of Diseases; APC: Annual percentage change; AAPC: Average annual percentage change.

## Competing interests

The authors declare that they have no competing interests.

## Authors’ contributions

AL and CS designed the methodology, retrieved and analysed the data. TS wrote the manuscript and conducted the joinpoint analysis. ND provided statistical advice on the use of regression techniques, reviewed and edited the manuscript. TM reviewed the manuscript and guided the discussion. All authors read and approved the final manuscript.

## Pre-publication history

The pre-publication history for this paper can be accessed here:

http://www.biomedcentral.com/1471-2377/14/2/prepub
